# Monitoring Circulation During Transition in Extreme Low Gestational Age Newborns: What’s on the Horizon?

**DOI:** 10.3389/fped.2018.00074

**Published:** 2018-03-26

**Authors:** David Van Laere, Michiel Voeten, John M. O’ Toole, Eugene Dempsey

**Affiliations:** ^1^Department of Neonatal Intensive Care, Antwerp University Hospital, Antwerp, Belgium; ^2^Laboratory of Experimental Medicine and Pediatrics, Faculty of Medicine and Health Sciences, University of Antwerp, Antwerp, Belgium; ^3^Irish Centre for Fetal and Neonatal Translational Research, University College Cork, Cork, Ireland

**Keywords:** hemodynamic, circulation, preterm, signal analysis, monitoring

## Abstract

Echocardiography and near-infrared spectroscopy have significantly changed our view on hemodynamic transition of the extreme preterm infant. Instead of focusing on maintaining an arbitrary target value of blood pressure, we aim for circulatory well-being by a comprehensive holistic assessment of markers of cardiovascular instability. Most of these clinical and biochemical indices are influenced by transition itself and remain poor discriminators to identify patients with a potential need for therapeutic intervention. At the same time, the evolution in data capturing and storage has led to a change in our approach to monitor vital parameters. Continuous trend monitoring has become more and more relevant. By using signal extraction methods, changes in trends over time can be quantified. In this review, we will discuss the impact of these innovations on the current monitoring practices and explore some of the potential benefits these techniques may have in improving real-time detection of extreme low birth weight infants at risk for morbidity related to impaired hemodynamic transition.

## Introduction

Transition from the fetal to the neonatal circulation is a complex and challenging process. What is considered as a small step for a healthy term infant, is a giant leap for a vulnerable preterm neonate. An increased understanding of the (patho-)physiology of hemodynamic transition combined with the digital revolution and increased monitoring storage capacity has opened the gate toward a more individualized approach. As current literature lacks robust evidence on treatment thresholds, an in-depth knowledge of the transitional physiology with a specific focus on the vulnerability of the preterm cardiovascular system is the cornerstone to guide these infants through their hemodynamic journey in the first days of life.

## The Physiology of Transitional Hemodynamic Adaptation

The fetal cardiovascular system has distinct morphologic and functional features that are different compared to the neonatal adapted circulation. The fetal myocardium consists of developing cardiac myocytes with an immature cell structure (1, 2). *In utero*, the right ventricle is the dominant chamber and both ventricles contribute to systemic blood flow to ensure adequate tissue oxygenation in a low oxygen tension environment. Both pulmonary and systemic circulation work in parallel through the presence of intra- and extra-cardiac shunts (foramen ovale, patent ductus arteriosus). As oxygen is delivered through the placenta, blood is diverted away from the pulmonary circulation by maintaining a high pulmonary vascular resistance (PVR). The placenta functions as a low resistance system making afterload suitable for the developing heart.

**Figure 1:**
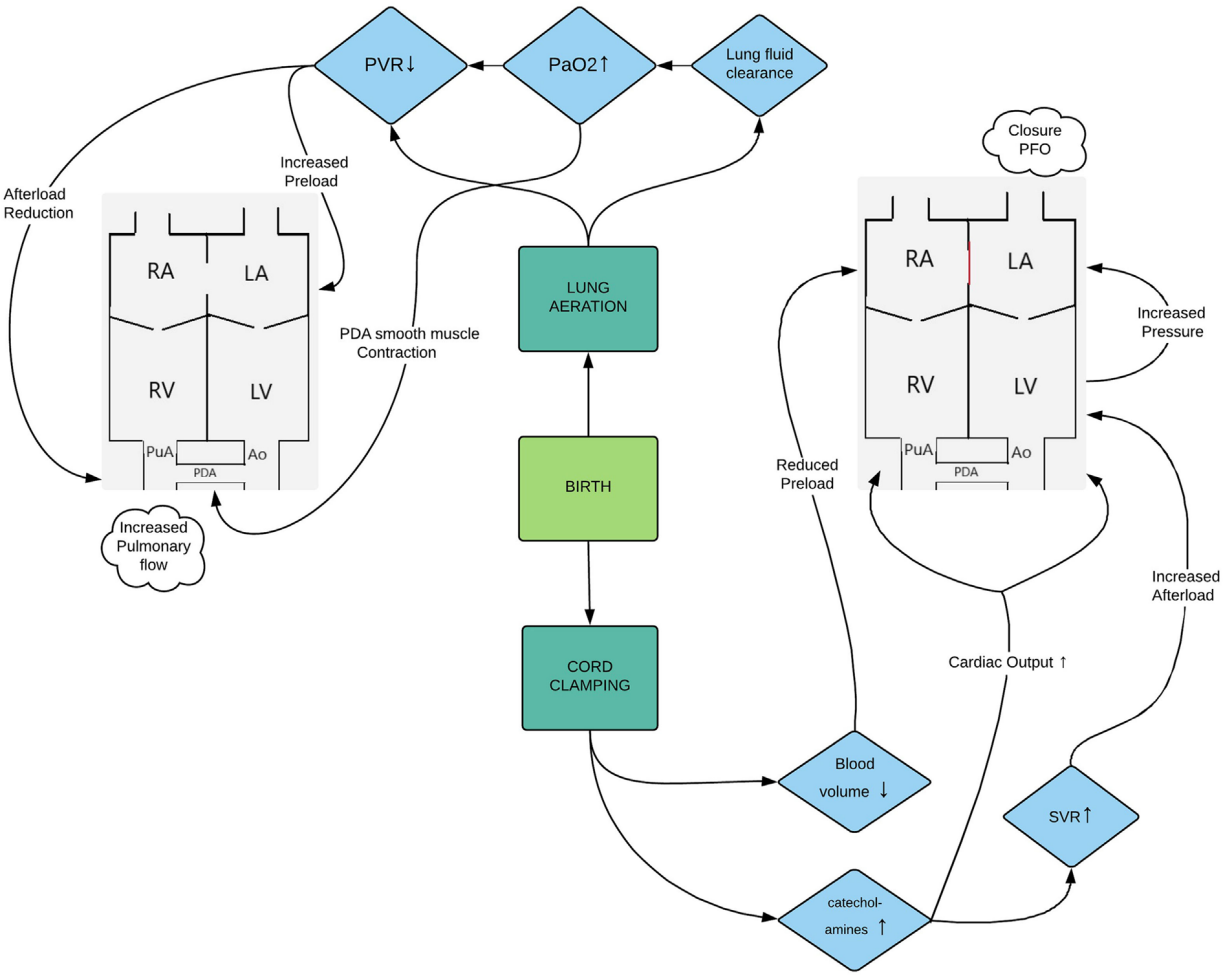
Flowchart representing the hemodynamic changes during transition from intra-uterine to extra-uterine life. Abbreviations: Ao, aorta; LA, left atrium; LV, left ventricle; PaO_2_, partial arterial pressure of oxygen; PDA, patent ductus arteriosus; PFO, patent foramen ovale; PVR, pulmonary vascular resistance; PuA, pulmonary artery; RA, right atrium; RV, right ventricle; SVR, systemic vascular resistance.

At birth, the partial pressure of oxygen rapidly rises in response to lung aeration causing a drop in PVR and a redirection of the right ventricular output toward the pulmonary circulation (3). The loss of the placenta leads to a sudden change in loading conditions of the preterm myocardium as the placenta functions as a low resistance system *in utero* and holds a substantial part of the blood volume (4). On the other hand, afterload increases as clamping of the cord leads to a surge of catecholamines (5) and other vasoconstrictor substances resulting in a rise in systemic vascular resistance. Figure 1 gives an overview of the hemodynamic changes during transition from intra-uterine to extra-uterine life.

## The Pathophysiology Related to Prematurity

As the myocardium is in an embryological stage of development, the preterm infant is at risk of hemodynamic compromise. Compared to healthy term newborns, systolic and diastolic performance of the myocardium seems to be impaired (6). Changes in loading condition, as is the case during the transitional period, might not be well tolerated by the preterm heart (7). The difference in cord clamping time and increased risk of prolonged exposure to fetal shunts may lead to a variable degree of volume loading. The relatively high resting heartbeat, the variable ontogenic expression of adrenergic receptors and the immaturity of the hypothalamic–pituitary axis limit the ability of the preterm heart to increase contractility (8, 9). The context of prematurity (inflammation, sepsis) itself and the level of intensive care treatment given at birth (ventilation, etc.) may also have an important impact on the cardiovascular response (10, 11).

To ensure normal cellular function, oxygen delivery must meet oxygen demand. This can only occur when oxygen content of the blood is ensured, and cardiac output and perfusion pressure are maintained. The most important clinical organ for impaired cardiovascular adaptation is the vulnerable preterm brain. Although multifactorial, studies with echocardiography have shown that transient reduced systemic blood flow plays a role in the pathophysiology of intraventricular hemorrhage (9, 12–14).

## A Pressure Driven Approach

Historically, a stringent approach based on mean arterial blood pressure was used to assess cardiovascular well-being during transition. This parameter is readily available and can be monitored continuously if an indwelling catheter is present. Although hypotension during the transitional period is associated with short-term adverse outcome (15), there is currently no evidence that treatment of low blood pressure improves outcome (16, 17). This might be related to an oversimplified approach in a complex clinical situation. The current thresholds for low blood pressure in neonates are statistically derived values based on studies done more than 20 years ago (18). The current cutoff values are the lowest centiles for blood pressure for a given gestational age, but they lack a physiological substrate at which disease occurs. Additionally, by using mean blood pressure, valuable information on the components of blood pressure is lost. Moreover, blood pressure is the dependent product of two independent variables: cardiac output and vascular resistance. During transition the correlation between systemic blood flow and blood pressure is weak (19). As such, the current cutoff values for hypotension have low sensitivity for detecting impaired cardiac output.

Recently a more pragmatic approach toward low blood pressure has found its way in many units. This consists of permissive hypotension if other hemodynamic parameters (heart rate, diuresis, lactate levels) are indicative of a state of cardiovascular well-being.

Randomized controlled trials are currently investigating whether a more stringent approach toward keeping blood pressure above a certain threshold is beneficial compared to an attitude of watchful waiting (15, 20).

## A Comprehensive Neonatal Hemodynamic Monitoring Model

An idealistic hemodynamic monitoring model that allows for an individual assessment of changes in the circulation would consist of real-time assessment of myocardial function and stroke volume on the one hand and information on peripheral and/or end-organ perfusion on the other hand. Over recent years new monitoring modalities have found their way to clinical practice (21, 22) making it possible to receive additional information on end-organ perfusion in a continuous and non-invasive manner. Increased digital storage capacity has made it possible to visualize trends of vital parameters over time, shifting the focus away from a single “golden bullet” cutoff value at which disease occurs in a group of patients. With advancing computing capacity and improved extraction methods it has become possible to decompose monitoring signals and to quantify their different characteristics. Interaction between different monitoring signals can be interrogated. Signal analysis may have the potential to detect patterns in vital parameters that are predictive of a potential pathologic state.

We propose a hemodynamic monitoring approach that would include information on cardiac output by a non-invasive cardiac output monitor (NICOM) and point of care ultrasound combined with additional information of “classical” vital parameters and real-time information on cerebral and peripheral perfusion by near-infrared spectroscopy (NIRS) and plethysmographic Perfusion index (PI) monitoring, respectively (Figure S1 in Supplementary Material). As point of care ultrasound is beyond the scope of this review, we will provide an overview of the literature regarding validation and evidence associated with outcome of the abovementioned monitoring techniques in the preterm population. In addition, we will discuss the potential implications in clinical care of signal analysis of these continuously monitored parameters.

### Non-Invasive Cardiac Output Monitor

Determination of blood flow through invasive techniques is an integral part of advanced hemodynamic monitoring in adult intensive care and perioperative medicine (23). NICOM is an emerging tool that allows for a continuous assessment of cardiac output. Currently available commercial monitors are based on bioimpedance (electrical cardiometry) or bioreactance technology. Cardiac output is derived by changes in either thoracic impedance or induced phase shift of an electrical current by pulsatile changes in blood volume (24). Validation studies in a preterm population have been performed. Left and right ventricular output measured by electrical cardiometry correlated significantly with echocardiographic measurements (25). There was no significant correlation with superior vena cava (SVC) flow. The limits of agreement were wide and decreased in the presence of high frequency ventilation and low output states, making the technique only feasible for trend monitoring. Another study of paired measurements performed in patients with a hemodynamically significant PDA showed similar findings (26). Reference normograms based on gestational age and birth weight were also recently published (27).

Non-invasive cardiac output monitor measurements of cardiac output based on bioreactance correlated well with left ventricular output on echocardiography in a study performed in term neonates (22). Readings of cardiac output, however, tended to under-read echo measurements. In a study performing paired measurements of bioreactance NICOM and echocardiography in preterm patients undergoing ligation showed that NICOM output measurements correlated well with stroke volume though there was a consistent underestimation with wide limits of agreement.

At present, no studies have been performed linking NICOM measurements with (adverse) outcome of prematurity.

### Peripheral Perfusion Monitoring: PI

Perfusion index is derived from the plethysmographic signal of a pulse oximeter. It is readily available on most monitoring systems in the NICU. The numerical value represents the ratio of light absorbed by pulsatile and non-pulsatile absorbers (28). As non-pulsatile absorbers are constant, the PI value is mainly influenced by arterial perfusion. Interestingly point measurements of PI during echocardiography correlated well with SVC flow in a study performed in preterm patients born before a gestational age of 32 weeks (29). A cutoff value of 0.44 had a negative predictive value of 98.6% for detecting low SVC flow during the first 72 h of life. Longitudinal reference values for PI during the first 72 h in patients born below a gestational age of 32 weeks have been published (30). The authors also presented the relationship of PI with clinical variables. PI was positively related with gestational age and negatively with mean arterial blood pressure and treatment with dopamine.

A recent study demonstrated that preductal PI and mean ΔPI of pre- and postductal measurements 4 h prior to echocardiography were able to identify preterm infants with a patent ductus arteriosus (31). PI values have been linked with outcome in both term and preterm infants (32, 33).

**Figure 2:**
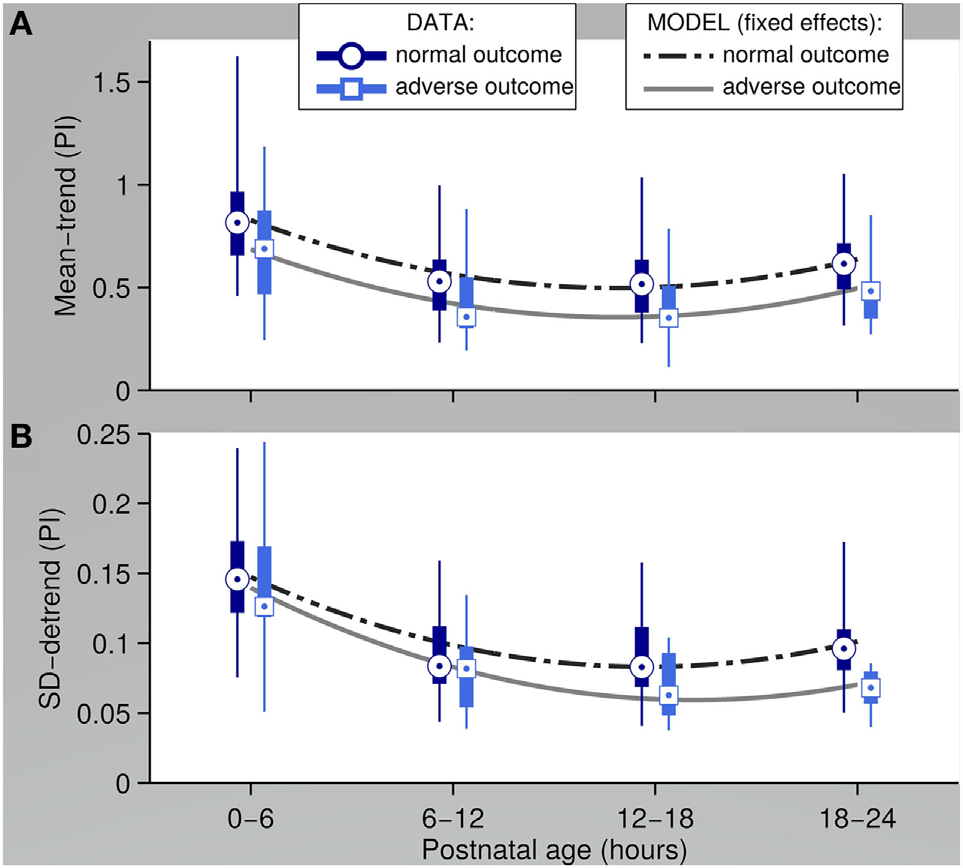
Perfusion Index (PI) features assessed over a 6-h interval with fixed-effects (outcome group) from the mixed effects model in a study performed in extreme low gestational newborns. Adverse outcome was defined as early mortality or acquired brain injury **(A)** mean of the PI trend **(B)** SD of the PI detrend [data adapted from Van Laere et al. (34)].

In a study using signal analysis extraction methods, the PI signal was separated into a low frequency (trend) component and a high frequency (detrend) component (34). Mean and SD were used as quantitative features to assess differences in temporal evolution of the components between patients with acquired brain injury or early mortality and patients with normal short-term outcome. Interestingly, patients with adverse outcome had significantly overall lower trend values for the first 24 h and decreased detrend variability from 12 h onward compared to patients with normal outcome (Figure 2). These effects remained significant when controlling for confounders such as gestational age and inotropic drug therapy. This study clearly shows that analyzing trend signals can identify subtle and clinically undetected changes in peripheral perfusion. The question remains if early identification and intervention can improve outcome.

### Cerebral Oximetry Monitoring: NIRS Monitoring

Near infrared spectroscopy technique is based on different wavelength absorption of near infrared light between oxygenated and deoxygenated hemoglobin. These differences are detected by a sensor and subsequently used to calculate the concentration of both types of hemoglobin. RcSO2 or Tissue oxygenation index represents the ratio of oxygenated to deoxygenated hemoglobin (35). As such, it represents a mixed saturation largely determined by the venous component. Different commercial devices are available and the technique is well validated in infants (36). Reference values based on large observational study in preterm infants born before 32 weeks of gestation have been published (37). RcSO2 values generally increase after birth and normal values range between 55 and 85% depending on gestational age. Small neonatal sensors have been developed and are now in use. Understanding the device and its values is essential as neonatal sensors generally overestimate values compared to adult sensors (38, 39).

Acquiring real-time information on cerebral oxygenation can be valuable for managing cardiorespiratory support during transition. While many extreme preterm infants are still ventilated, arterial carbon dioxide pressure can affect cerebral blood flow, where hypocapnia induces cerebral arterial vasoconstriction and hypercapnia induces vasodilation. A trial that randomized patients to visible reading of regional saturation with adherent treatment guidelines versus a control group blinded to these measurements showed a significant reduction in the burden of cerebral hypoxia and hyperoxia (40). The trial was not powered to show differences in clinical outcome. Interestingly a *post hoc* analysis showed an association with lower early burden of hypoxia and severe brain injury of prematurity (41). More recently a feature set of the RcSO2 signal was developed for the purpose of detecting brain injury. Cross validation was performed in a cohort of infants <32 weeks of gestation. Although some features were significantly correlated with brain injury, sensitivity and specificity were moderate. Nevertheless, the quantitative analysis of the rcSO2 signal might contribute to early identification of infants at risk (42).

While continuous cerebral oxygenation monitoring can assess the real-time effect of interventions on brain perfusion, the most intriguing innovation comes from the ability to assess whether changes in the invasive blood pressure signal correlate with similar changes in the NIRS signal. This parameter can then be used as a surrogate measurement for cerebral autoregulation. The debate is still ongoing on whether cerebrovascular autoregulation is developmentally and postnatally regulated and how illness severity and concomitant treatment interact with it. Different techniques have been developed to quantify cerebral autoregulation. In essence, the relationship between both signals can be assessed in the time or frequency domain. Another technique suggested an approach using moving correlation between heart rate and NIRS derived variables, making it feasible for patients without an indwelling arterial line (43). A recent extensive review gives an in-depth overview on the current literature on this topic (44). Several studies with different techniques found a significant association with increased markers of impaired autoregulation and acquired brain injury of prematurity (43, 45–48). Providing real-time quantitative information on cerebral autoregulation could potentially imply that the clinician becomes aware of an individual safe range of blood pressure. At present, upper and lower thresholds of arterial blood pressure at which cerebral blood flow becomes pressure passive remain largely unknown due to a lack of consensus on the most robust methodology to assess cerebrovascular autoregulation and which outcome to measure.

## Future Perspectives: Toward an Individualized Approach

In the last decade, non-invasive monitoring tools have been developed and some of them already found their way to the bedside. Improved storage and computing capacity has made trend monitoring more feasible and attractive. Combining multiple parameters and quantifying signal characteristics make it possible to detect or predict disease in a subclinical phase. Monitoring heart rate characteristics for early detection of sepsis has shown to be associated with a reduction of septicemia related mortality (49). Technologies for automated seizure detection are currently being evaluated for additive diagnostic accuracy at the bedside (50).

At present, there is paucity of evidence on how treatment adherent to new parameters and innovative techniques can improve clinical outcomes in the preterm population during transition. Nevertheless, technological advancement has provided more insight in transitional physiology and the challenges for each individual preterm infant. The next step will be to incorporate real-time bedside signal processing monitors into clinical trials. As we are slowly but surely moving away from the era of pragmatic cutoff values for generalized treatment, a new individualized approach to treatment will pose a big challenge to the evidence-based neonatal community. Large collaborative networks on continuous data with input from biomedical engineers and computer scientist are the way forward to overcome these issues. We are hopeful that the next phase will be to feed monitoring algorithms with proven clinical usefulness into a cognitive computing model to develop a predictive monitoring system.

## Author Contributions

DL conceptualized this review and wrote the first draft and revised the manuscript for submission. All the other authors critically reviewed the manuscript. All authors approved the final manuscript as submitted and agreed to be accountable for all the aspects of the work.

## Conflict of Interest Statement

The authors declare that the research was conducted in the absence of any commercial or financial relationships that could be construed as a potential conflict of interest.

## Supplementary Material

Figure S1 Photo of manikin representing a hemodynamic monitoring model in an extreme preterm infant.

## Abbreviations

ELGANs, extreme low gestational age newborns; NICOM, non-invasive cardiac output monitor; NIRS, near-infrared spectroscopy; PI, perfusion index; PVR, pulmonary vascular resistance; SVC, superior vena cave.
