# Supplementary figures and images for: Monitoring Circulation During Transition in Extreme Low Gestational Age Newborns: What’s on the Horizon?
Source: Front Pediatr. 2018 Mar 26;6:74. doi: 10.3389/fped.2018.00074 (PMC5879103; doi:10.3389/fped.2018.00074)

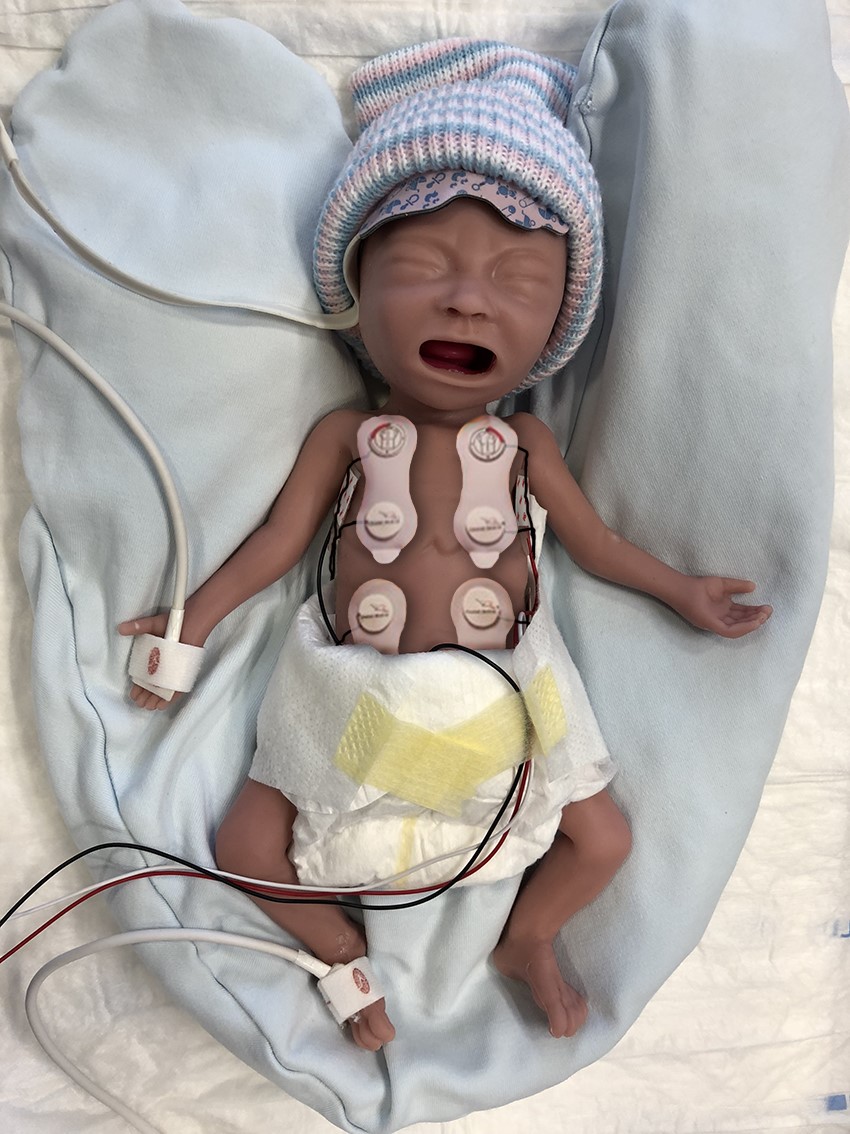

Supplement: Figure S1 — Photo of manikin representing a hemodynamic monitoring model in an extreme preterm infant. [file image_1.jpeg]
